# Supplementary material for: Characterization of Phytohormones and Transcriptomic Profiling of the Female and Male Inflorescence Development in Manchurian Walnut (Juglans mandshurica Maxim.)
Source: Int J Mol Sci. 2022 May 13;23(10):5433. doi: 10.3390/ijms23105433 (PMC9143237; doi:10.3390/ijms23105433)
Supplement: Supplementary file 1 [file ijms-23-05433-s001.zip › Supplementary Figures.pdf]

Supplementary Figures

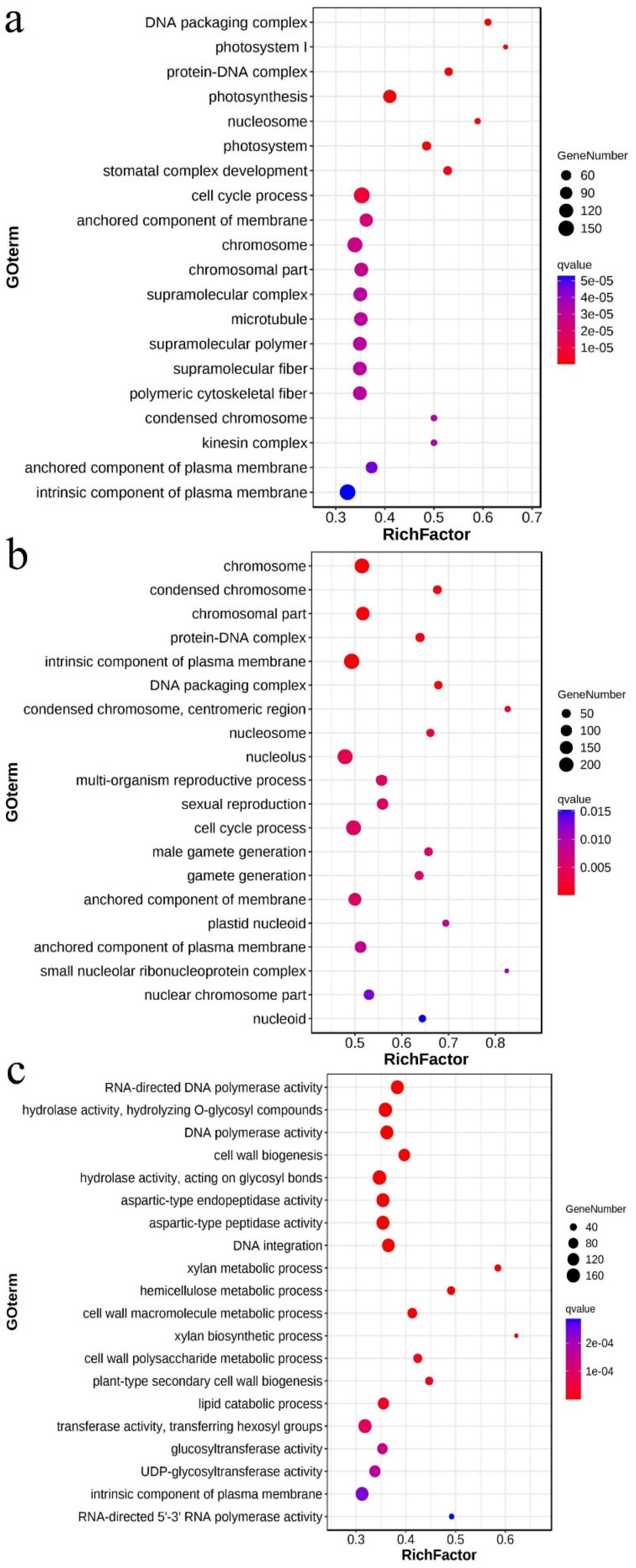

Figure S1 The GO enrichment analysis of differentially expressed genes during male inflorescence development. (a-c) indicate the top 20 enriched GO terms of the DEGs in MS1 vs MS2, MS1 vs MS3 and MS2 vs MS3, respectively.

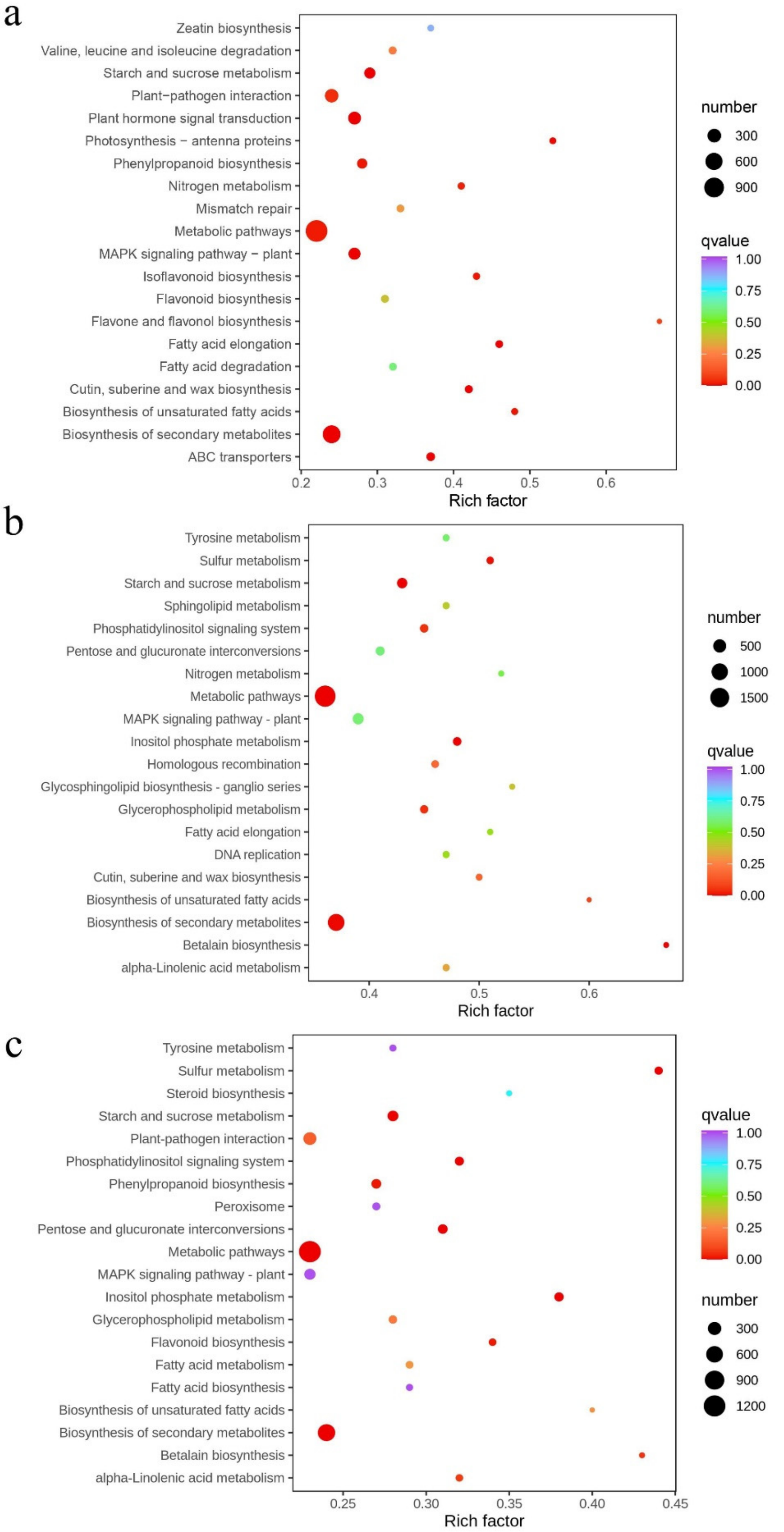

Figure S2 The KEGG enrichment analysis of differentially expressed genes during male inflorescence development. (a-c) indicate the top 20 enriched GO terms of the DEGs in MS1 vs MS2, MS1 vs MS3 and MS2 vs MS3, respectively.

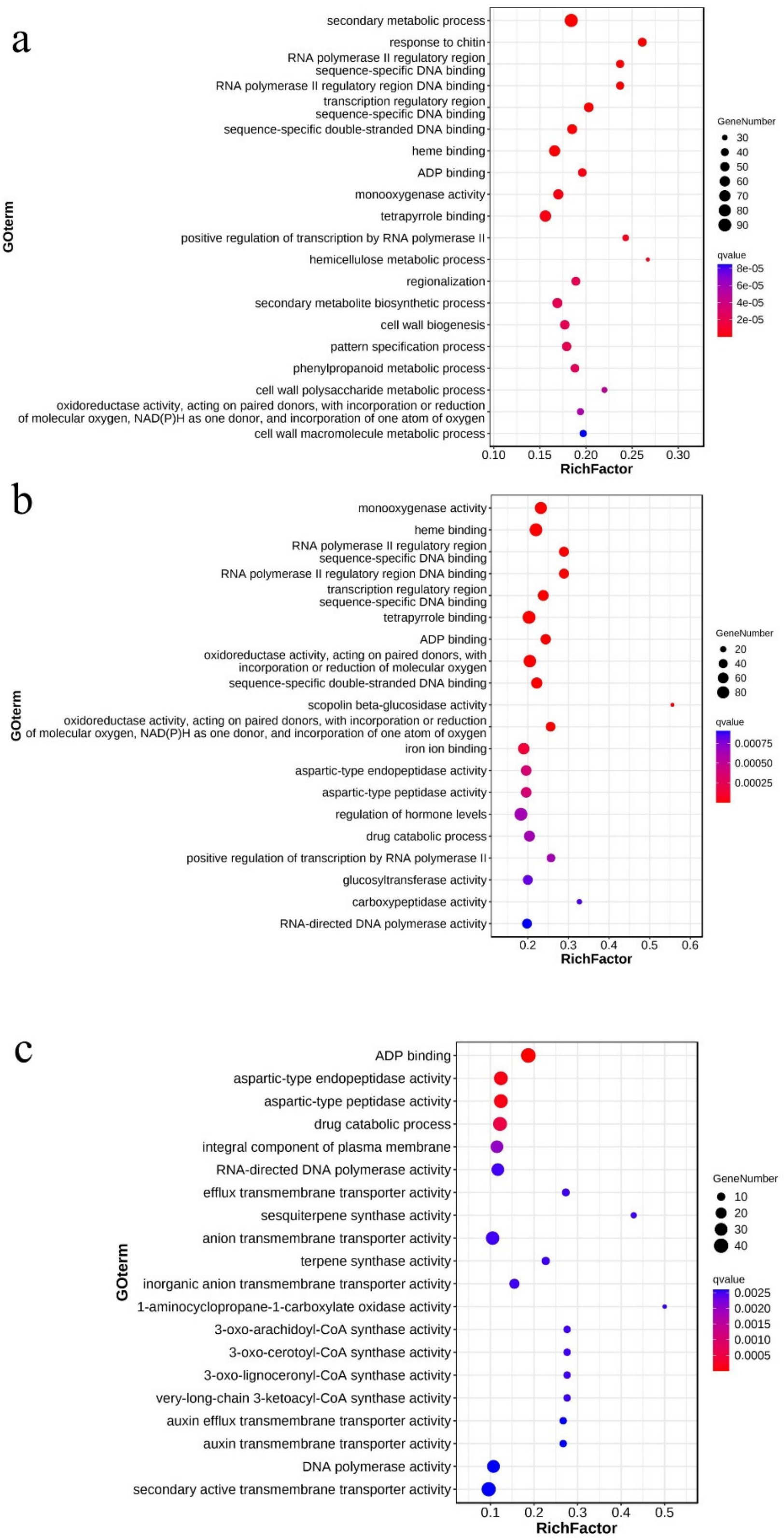

Figure S3 The GO enrichment analysis of differentially expressed genes during female inflorescence development. (a-c) indicate the top 20 enriched GO terms of the DEGs in FS1 vs FS2, FS1 vs FS3 and FS2 vs FS3, respectively.

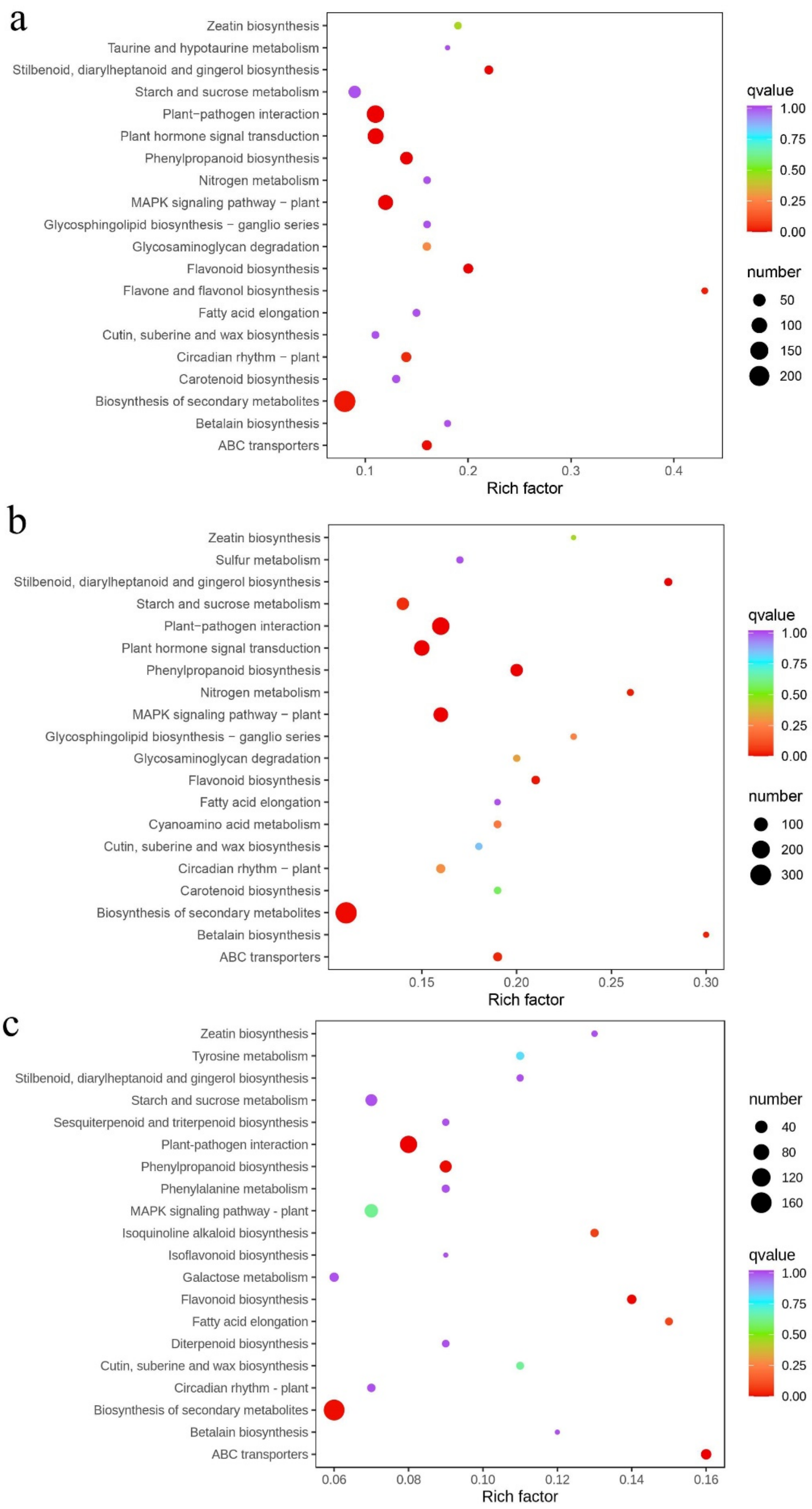

Figure S4 The KEGG enrichment analysis of differentially expressed genes during female inflorescence development. (a-c) indicate the top 20 enriched GO terms of the DEGs in FS1 vs FS2, FS1 vs FS3 and FS2 vs FS3, respectively.
